# A Simulation-Based Methodology of Developing 3D Printed Anthropomorphic Phantoms for Microwave Imaging Systems

**DOI:** 10.3390/diagnostics11020376

**Published:** 2021-02-22

**Authors:** Soroush Abedi, Nadine Joachimowicz, Nicolas Phillips, Hélène Roussel

**Affiliations:** 1 Sorbonne Université, CNRS, Laboratoire de Génie Electrique et Electronique de Paris, 75252 Paris, France; nadine.joachimowicz@paris7.jussieu.fr (N.J.); helene.roussel@sorbonne-universite.fr (H.R.); 2 Université de Paris, IUT, 20 quarter rue du département, 75018 Paris, France; nicolas.phillips@etu.utc.fr

**Keywords:** microwave imaging, anthropomorphic standardized biological phantom, stroke monitoring, tumor detection, head phantom

## Abstract

This work is devoted to the development and manufacturing of realistic benchmark phantoms to evaluate the performance of microwave imaging devices. The 3D (3 dimensional) printed phantoms contain several cavities, designed to be filled with liquid solutions that mimic biological tissues in terms of complex permittivity over a wide frequency range. Numerical versions (stereolithography (STL) format files) of these phantoms were used to perform simulations to investigate experimental parameters. The purpose of this paper is two-fold. First, a general methodology for the development of a biological phantom is presented. Second, this approach is applied to the particular case of the experimental device developed by the Department of Electronics and Telecommunications at Politecnico di Torino (POLITO) that currently uses a homogeneous version of the head phantom considered in this paper. Numerical versions of the introduced inhomogeneous head phantoms were used to evaluate the effect of various parameters related to their development, such as the permittivity of the equivalent biological tissue, coupling medium, thickness and nature of the phantom walls, and number of compartments. To shed light on the effects of blood circulation on the recognition of a randomly shaped stroke, a numerical brain model including blood vessels was considered.

## 1. Introduction

Microwave technology offers a low-cost, mobile, and non-ionizing diagnostic alternative modality for applications such as cerebrovascular disease monitoring or tumor detection. Several studies have shown that, in the microwave frequency range, a significant difference in dielectric properties exists between normal and pathological tissues [1,2,3,4]. Consequently, several research teams are working on this topic around the world and some imaging systems dedicated to these applications are emerging [5,6,7,8,9]. The development of such imaging devices should help to improve prehospital diagnostic accuracy which is essential to decrease treatment time, thereby increasing survival and mitigation of injury [10].

For evaluation of these imaging devices, a set of experimental data is needed. They should be collected in controlled configurations on clinical trials. Clinical trials are the best way to assess devices and some groups are active in this domain for stroke detection for example [7,8,9,10]. However, for several reasons, it is preferable, in the first step of development, not to employ the human body for testing microwave imaging devices, as they have electromagnetic (EM) interaction with the human body. Besides safety reasons, respiratory movement, cardiovascular vibration, variable skin humidity, and ethics approval can be mentioned as obstacles in using human tissues first of all in imaging systems. Therefore, there is a need for stable and realistic reference phantoms. On that point, a clear validation of the performance of these phantoms is needed even though they can be difficult to customize.

Emulating a human organ with an exact substitute requires an accurate artificial phantom. This phantom is expected to ideally have the following features: (1) reasonably realistic anatomy, (2) a long lifetime (robust and stable), (3) easily adjustable to any experimental set-up, (4) to be safe, and easily and remotely reproducible, (5) to be able to perform simulations along with experimental validations, (6) low cost, and (7) dielectric precision across a wide frequency range if it is used for microwave imaging. 3D printing is a rapid prototyping technique that is used widely and is ideal for quickly producing customized, low cost, and reusable imaging phantoms [11]. Burfeindt et al., have given the scientific community access to an STL file of a breast phantom [12] made of a unique cavity. This gave rise to the 3 cavities GeePs-L2S breast phantom [13] which is designed and developed by using CAD software from the STL file in open access and whose printed form was in turn shared within the framework of COST TD1301 MiMed to test several microwave imaging systems in development [14,15,16,17,18].

The paper is organized as follows. In the second section, the methodology of developing 3D printed phantoms filled with mixtures mimicking tissues, for assessment of microwave imaging is presented. After a non-exhaustive literature review of different kinds of phantoms that used 3D printing technology, the general concept suggested in the paper of developing anthropomorphic phantoms for microwave imaging is illustrated by a flowchart in Section 2.1. Section 2.2 is devoted to the fabrication and characterization of 10 tissue-mimicking materials (TMMs) based upon Triton X-100 and salted water, whose compositions are predetermined by using Böttcher [19,20] or the Kraszewski binary mixture law [21]; the use of the latter, as well as the experimental validation of the numerical results obtained in [20], constitutes a new development introduced in this paper. In Section 3, the process is applied to a head phantom developed in the frame of the EMERALD project [6]. The head phantom described in Section 3.1 is built up from the STL format file [22] obtained by Computer-Aided Design (CAD) software. Its initial version has already been described in [19] and its upside-down homogeneous version is used in the experimental set-up presented in [5]; now it has two options of the brain (simple or complex brain shape) and additional cavities. Section 3.2 gives the composition of the liquid mixtures used to fill up the cavities. Finally, Section 3.3 is devoted to the computation of the total field distribution inside the anthropomorphic head phantom by using its numerical version (the STL file). This phantom is surrounded by a conformal helmet including 24 monopole antennas. Thus, the effect of the coupling medium, the ABS phantom structure, and the design are discussed in the frame of the device developed in [5].

## 2. Materials and Methods

In recent years, some researchers used 3D printing technology to make phantoms. In addition to surgical planning applications, 3D printed phantoms are essential for the validation of medical computational models, as well as for medical training and patient education. In [23] there is a comprehensive recent review of the state of the art along with new developments and trends in 3D printed functional medical phantoms (i.e., tissue-mimicking medical phantoms, radiologically relevant medical phantoms, and physiological medical phantoms) and 3D bio-printed structures (i.e., hybrid scaffolding materials, convertible scaffolds, and integrated sensors) for regenerated tissues and organs. There is another research on 3D printed tumors that aids researchers in the study of metastasis and leads to facilitating complex treatment, surgery, and therapies [24]. In this way, 3D printing can significantly improve patient comfort and treatment accuracy [25].

To obtain realistic phantoms it is crucial to get as close to the anatomy by using Magnetic Resonance Imaging (MRI) or Computed Tomography (CT) scans to develop phantoms for imaging with MRI, CT, positron emission computed tomography (PET), single-photon emission computerized tomography (SPECT), and ultrasound [26]. In the microwave imaging domain, anthropomorphic head phantoms were developed by printing 3D molds intended to be filled with gel-based parts of the head [27,28] or mixtures based on graphite carbon black polyurethane [29,30]. A review of the development of anthropomorphic breast phantoms is given in [31].

As defined in the framework of the EMERALD project, the manufacturing process used to build up accurate ultra-wideband (UWB) phantoms has to be easily reproducible by an electrical engineer in a non-specific environment without extreme precautions, and to some extent at low cost. The specificity of the GeePs-L2S breast phantom and those suggested in our previous and current works on the head [19,32], breast [13,33], and thorax [34,35] phantoms, has been confirmed by the work of [14,15,16,17,18] and [36,37,38,39]. These phantoms composed of several 3D printed cavities are filled up with liquid mixtures made for example of Triton X-100 (TX-100, a non-ionic surfactant) and salt water, the concentrations are numerically adjusted so that the dielectric properties are close to the reference values over a wide frequency range. Besides, not only liquid mixtures are adjustable over time, but also the mixture of the components mentioned above which provides stability of dielectric properties and state of matter over time. Finally, the numerical version of the phantom (STL file) offers remote reproduction and can be used to perform simulations to test experimental parameters and improve the device. Satisfying the seven criteria listed in the introduction makes these phantoms appropriate for the framework of projects such as EMERALD.

### 2.1. Flowchart

**Figure 1:**
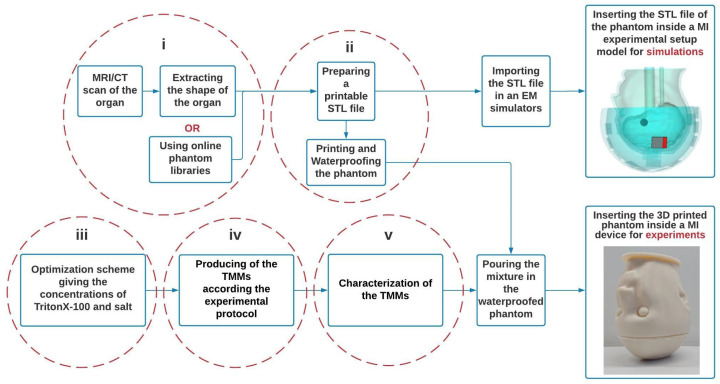
Three-dimension (3D) Printed benchmark phantom flowchart for microwave imaging.

Here in a flowchart (see Figure 1) the process of developing benchmark phantoms for the assessment of microwave devices is explained. 

(i) The STL files of the organs are obtained from the segmentation of an MRI/CT scan or a library of phantoms. 

(ii) The files are modified with a CAD software to develop printable cavities in such a way that the resulting phantom is fillable and compatible with the experimental system. Thus, the STL files should be provided in a way to be readable by 3D printers. 

(iii) TMMs are developed for each tissue according to the concentrations of TritonX-100 and salt determined from the optimization process.

(iv) The mixtures are prepared following the experimental process described in Section 2.2.2.

(v) Dielectric properties of the mixture are measured. Note that at this step, an experimental adjustment may be necessary to obtain properties closer to the theoretical values of less viscous mixtures.

The resulting phantom, which has the shape of the organ, is filled with TMM and can then be used for the evaluation of microwave imaging systems by being inserted into it; its digital version can be imported into EM simulators such as the CST Studio Suit^e®^ and WIPL-D^®^ [40] for electromagnetic simulations, to investigate the device as well as the phantom itself.

### 2.2. Tissue Mimicking Material (TMM) for 10 Biological Tissues

#### 2.2.1. Introduction

TMMs can be classified according to their physical appearance while each type has its benefits. Tissues with water content, such as most of the brain parts, have high dielectric properties. Therefore, using water as one of the components of TMM seems to be reasonable. In this case, using liquid TMM will be suitable and easy to produce. For low permittivity tissues like bone and fat, liquid TMM can still be used by decreasing the percentage of water and adding liquid components that have low permittivities such as glycerin [41], sugar [42], and Triton X-100 [13]. There are also gel-based TMMs that are more similar to solid mixtures but, as with liquid mixtures, they require the manufacture of containers. Several gel-like materials have been proposed in the literature for different body parts, such as for the muscle [43], brain [44], and breast [45,46]. On the contrary, solid TMMs are not water-based. Graphite powder and ceramic are some examples of different ingredients used to produce solid TMMs [29,30,47].

Using 3D printing technology to design a container with a realistic shape of the aimed organ and filling it with a liquid mixture mimicking the dielectric properties of the organ, gives us the chance to have a reproducible, adjustable, and reusable phantom that none of the other types of phantoms can offer. As it is described in [19] the composition of the mixture is simple: TritonX-100 and salt water. This results in a binary liquid mixture that can be modeled by a mixing law.

#### 2.2.2. Development

- Optimization Scheme

We assume that the TX100-saline water mixture is a binary mixture, for which Debye’s model for the Triton X-100 (group 3 [48]) and that for saltwater [49] are known. Thus, the complex permittivity of the mixture can be obtained using a binary law of liquid mixing. Among the different models, which can be found in the literature [50], five formulas have been evaluated: Lichtenecker, Looyenga, Bruggman, Böttcher, and newly Kraszewski. Only the Böttcher and Kraszewski formula give permittivity values similar to those measured, as described in Section 2.2.3, for different concentrations of Triton X-100 and salt, on a wide frequency range. Since the study with the Böttcher formula was reported in [19], the procedure will be described herein using the Kraszewski formula, which reads as follows:

(1) $$\varepsilon_{m}={\left[\varepsilon_{1}^{1/2}+V_{2}\left(\varepsilon_{2}^{1/2}-\varepsilon_{1}^{1/2}\right)\right]}^{2}$$

ε is the complex permittivity where subscripts 1 and 2 stand for Triton X-100 and salt water, respectively. $V_{2}$ denotes the volumetric proportion of salt water in the mixture while the respective volumes of the latter are such that $V_{1}+V_{2}=1$.

Parameters used in Cole–Cole models can be defined to describe the complex permittivity of human tissues as a function of frequency [51]. Hence, the Triton-X and salt concentrations need to mimic a specific tissue that can be computed by fitting the complex permittivity of the mixture model $\varepsilon_{m}$ to the one of the tissue $\varepsilon_{t}$ over a specific frequency range. This is done by using a Gauss–Newton process [52], where at each iteration, the NaCl concentration and volume fraction of Triton-X are determined at discrete frequencies over a frequency range [500 MHz to 3 GHz], by minimizing the cost functional $J=\;\underset{f}{\sum }\omega_{f}{\left|\varepsilon_{m}-\varepsilon_{t}\right|}_{f}^{2}$, where $\omega_{f}=1/{\left|\varepsilon_{t}\right|}_{f}^{2}$.

The quadratic cost function is approached by its first (gradient **g**) and second (approximate Hessian **H**) derivatives with respect to the NaCl concentration and Triton-X volume fraction, which are computed analytically.

(2) $$g=2\sum_{f}\omega_{f}ℜe{\left[{\left({ℰ}_{m}-{ℰ}_{t}\right)}_{f}^{*}\overset{´}{{ℰ}_{m}}\right]}_{f}$$

(3) $$H=2\sum_{f}\omega_{f}ℜe{\left(\varepsilon_{m}^{’*}\varepsilon_{m}^{’†}\right)}_{f}$$

In the above equation $\varepsilon_{m}^{’}={\left(\partial \varepsilon_{m}/\partial V_{2}\;,\;\partial \varepsilon_{m}/\partial S_{m}\right)}^{†}$ where $S_{m}$ indicates the NaCl concentration of the mixture, and † the transposition.

Details are given in [19] for the Böttcher law, they are rewritten herein when the Kraszewski formula is used. Then by considering Equation (1), $\varepsilon_{m}^{\text{’}}$ becomes:

(4) εm’=(2((ε1ε2)1/2−ε1)+2V2(ε1−2(ε1ε2)12)+ε2)((ε1ε2)1/2+V2[1−(ε1ε2)1/2])∂ε2/∂S2),

As it is mentioned in [19] the term $\partial \varepsilon_{2}/\partial S_{2}$ can be inferred from the salted water parametric model.

At iteration step k + 1, the solution $x={\left(V_{2},S_{m}\right)}^{†}$ reads:

(5) $$x^{k+1}=x^{k}-H^{-1}\left(x^{k}\right)g\left(x^{k}\right),$$

This process converges towards a stable solution in a few iterations, most of the time independently of the initial NaCl concentration and Triton-X volume fraction, by inverting at each iteration, the approximate Hessian matrix **H** of rank 2.

- Experimental protocol

Following one of the objectives of the EMERALD project, the process of preparing the mixtures is easily reproducible by an electrical engineer in a non-specific environment. Here the protocol is explained step by step:

- Tare the balance while the empty beaker is upon the balance.
- The masses of NaCl and deionized water should be added successively considering that taring during the adding process is necessary.
- Using a magnetic bar for stirring will result in a homogenous solution. Tare the balance.
- Add the mass of TritonX-100. Heat up TritonX-100 in a 45 ° C water bath since the liquid is very viscous and needs to be fluid.
- Use the magnetic bar again while the solution is in a hot water bath to obtain a homogeneous mixture.
- Keep the solution in a container at room temperature and away from light for its conservation.

#### 2.2.3. Dielectric Characterization

The use of the Böttcher’s law in the optimization algorithm allowed numerical adjustment of the concentration of TX-100 and salt, required to produce TMMs with dielectric properties similar to 10 biological tissues, at room temperature, over a wide frequency range [20]. New similar results have since been obtained with the Kraszewski’s law (substantially similar solution and convergence).

**Figure 2:**
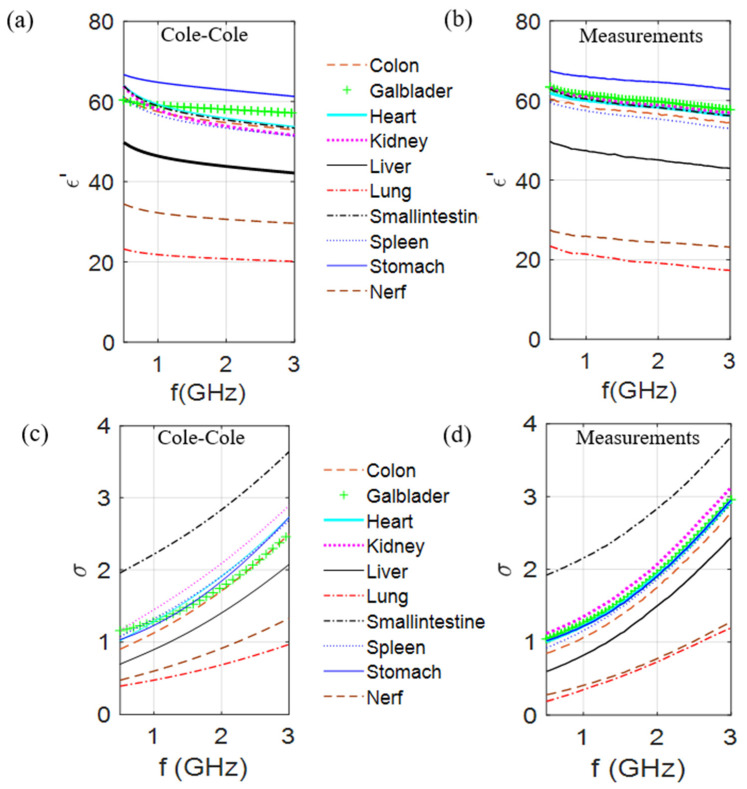
Comparison of the dielectric constant and conductivity of 10 different biological tissues of the values obtained from the Cole–Cole model (**a**,**c**) with the measured values (**b**,**d**) of produced mixtures at room temperature, over the [500 MHz to 3 GHz] frequency range.

From the results of the optimization process when the Böttcher [19] or the Krasweski binary laws were used, 10 mixtures were produced and characterized. The dielectric characteristics of these mixtures were measured by using an open-ended coaxial probe coupled to a Rohde & Schwarz ZVB8 vector network analyzer. The calibration process is based on the procedure, presented in [53]. Figure 2 gives the dielectric constant and the conductivity of the 10 biological tissues at room temperature over the [500 MHz to 3 GHz] frequency range; the first column, which corresponds to the reference values obtained from the Cole–Cole models, is compared with measured values of the developed mixtures (second column).

Results presented in Figure 2 validate the generalization of the process for any biological part of the body and show that it is possible to make mixtures mimicking the dielectric contrasts between the different tissues. In fact, taking into account that these measurements are dependent of the conditions by which they are made (in vivo/in vitro), a comparison on the dielectric contrast between curves seems to be more relevant. Following the proposed method, these results can be reproduced in different labs and obtain same variations of the complex permittivities as a function of the frequency. However, it is noteworthy that the TX-100–salt water mixtures are significantly easy to produce, except when the TX-100 volume percentage is approximately in the 40−60% range since the mixture is rather viscous at 25 °C. This is the case for the mixture mimicking the nerve tissue (44% TX-100) and as a result the deviation between the measured and Cole–Cole values is the highest (Figure 2). This phenomenon decreases by increasing the temperature and amount of salt. Indeed, very few TMM are concerned with this problem at room temperature. However, for the latter, the mixture components have to be warmed separately, then mixed, vigorously stirred, and left to rest at 45 °C for a few minutes until air bubbles vanish. Note also that for those cases, the quantity of salt and TX100 can be adjusted experimentally to obtain a liquid mixture that has dielectric properties close to the expected values at the given temperature.

## 3. Results and Discussion

### 3.1. Description of the Test Case

**Figure 3:**
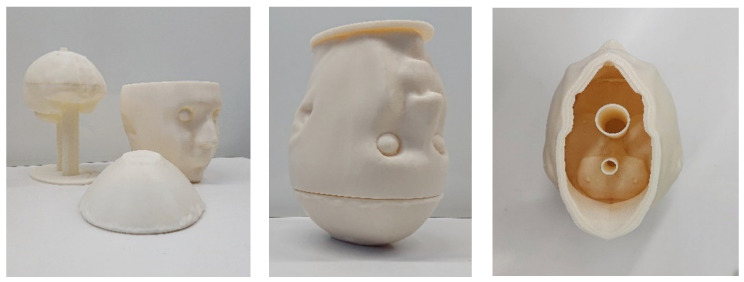
Upside-down configuration of the head phantom including the simple brain cavity.

**Figure 4:**
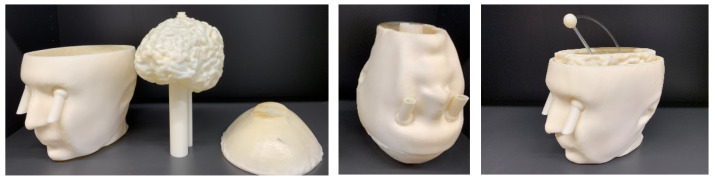
Upside down head phantom including the complex brain and an extra cavity as a muscle container. The stroke and the connected pipes are designed in a way to pass through the cylinders which are connected to the brain cavity and can be fixed in a random location inside of it.

The head phantoms are composed of ABS cavities filled with mixtures that mimic dielectric properties of Cerebrospinal Fluid (CSF), brain, blood (for the stroke cavity), and muscle for the additional cavity. Using two cylinders connected to the brain cavity (Figure 3), one can fill the latter and put any object inside it (as is shown in Figure 4) for testing microwave imaging systems and imaging algorithms. The brain is cut into two slices in such a way that the inner part is accessible to be cleaned after each experiment and the position of the stroke can be easily fixed. The phantom is designed in a way that at least two pieces engage and fit firmly, yet be easily disassembled, like a Lego piece, to prevent leakage. The designed phantom is upside down to be adapted to the microwave imaging system designed in POLITO [5]. To reach the space between the outer shell and the brain cavity one needs to remove the plate and this space can be filled easily with a mixture with the dielectric properties of the CSF. The plate stabilizes the position of the brain cavity since two cylinders go through it and also keeps the mixture inside of the phantom secure.

Figure 4 shows the 3-cavity version of the head phantom which has an additional cavity in the lower part, designed to be filled with a liquid mixture mimicking, for example in this paper the muscle tissue. Embedding two tubes in the eyes enables us to fill the CSF cavity, while by removing the plate the muscle cavity can be filled. The brain cavity is also changed to a complex version with circumvolutions. This new version of the brain is also supported by the 2-cavity phantom and can be replaced by the simple brain. The complex brain cavity is more realistic, but considering that it is made of plastic, it has a higher effective thickness. The effect of plastic on the distribution of the electric field is discussed later in the paper. 

### 3.2. TMM

Table 1 groups the results at 1 GHz and 25 °C for the head TMM involved in the next simulations. In this table, the brain is considered as a blend of white and grey matter (75% of white matter and 25% of grey matter) and “bone” refers to the cortical bone. Table 1 presents Triton-X and salt concentrations based on Kraszewski’s and Böttcher‘s binary laws over the range of 0.5 to 3 GHz. Moreover, the numerical values versus the expected ones (Cole–Cole, Ref [51]) for each tissue at 1 GHz are included. Results obtained from Böttcher’s and Kraszewski’s laws appear to be roughly equivalent for modeling TX100 and saltwater mixtures, providing access to a range of values for the experimental validation, no matter what the TX100 concentrations are (the same dielectric properties and the same convergence have been observed). Providing a range of values given by different binary laws, is important since first the experiments never fit the theory and second for specific concentrations of TX100 the solution will be a gel at room temperature which is not desired. Considering the concentrations of biological tissues presented in [19] at 37 °C, one can see a slight difference for the head mixtures despite the change of temperature, possibly except for the brain, for which the TX concentration is close to the viscosity limit and which can be viscous at room temperature.

### 3.3. Numerical Simulations of the Electric Field Inside and Outside the Head Phantom—Monopole Antenna Excitation

For all the results presented below, the computation of the electromagnetic field (EM) distribution inside and outside the phantoms, is conducted using the commercial software CST Microwave-Studio (time-domain solver). 

**Figure 5:**
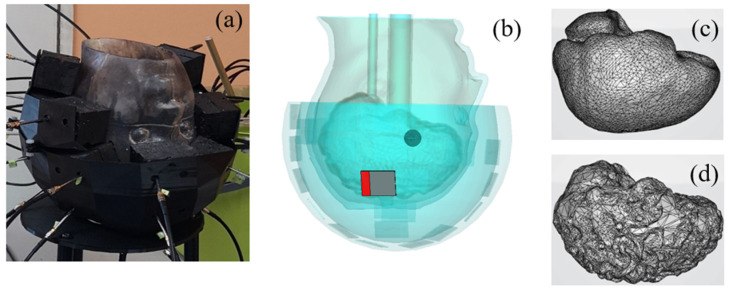
The imaging system [5], including a head phantom with a stroke mimicking anomaly and a 24-antenna array conformal to the head (**a**,**b**). Two different models of the brain are used: a simple (**c**) and a complex with convolutions (**d**).

Figure 5 displays the 3D head phantom with a stroke, placed inside a microwave imaging system inspired by the one described in [5], and two models of the brain. The head phantom is covered by a conformal helmet made of ABS (whose relative permittivity and conductivity are 3 and 0.004 S/m, respectively at 1 GHz) and of 24 implemented monopole antennas. Further details on the choice of the antenna array (arrangement and number of antennas, the working frequency and the matching medium), as well as on the brick-shaped antenna module used in the POLITO imaging system are given in [54] and [55] respectively. The influence on the E-field distribution, obtained when one of the 24 antennas (the one shown in red in Figure 5b) is active, is studied by varying parameters such as the coupling medium, chosen with low losses and a dielectric constant of 23 and the printed material (ABS), studied in Section 3.1, which is reported controversially in [56].

Some of the presented results from CST simulations are normalized as follows:

(6) $$Normalized\;Module=\left(Module-C_{min}\right)/\left(C_{max}-C_{min}\right),$$

where Module is the magnitude of total E field (the three components), $C_{max}$ and $C_{min}$ are the max and min values. The range of the color bar used to display the normalized electric field distribution is given between −10 to −40 dB while the differential field distribution is presented in volts per meter.

#### 3.3.1. Effect of the Coupling Medium

**Figure 6:**
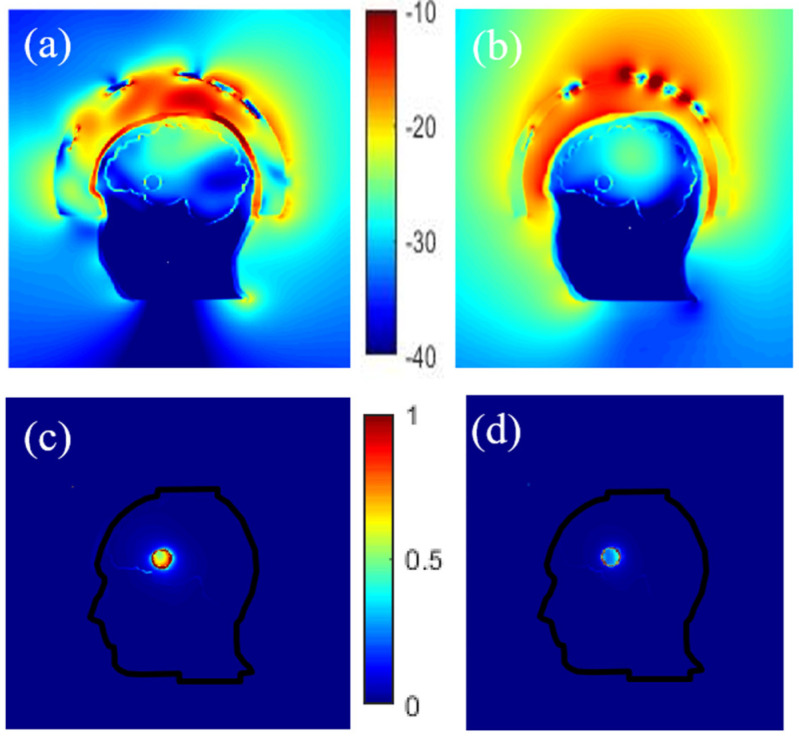
Normalized electric field magnitude distribution inside and outside the head for 2 different coupling mediums, TX-100/salted water (**a**) and air (**b**). The corresponding differential field distribution, TX-100/salted water (**c**) and air (**d**) respectively.

The magnitude of the electric field obtained inside the phantom for two different coupling mediums (interface between the antennas and the head) is given in Figure 6a,b. It indicates that a better penetration of the E-field into the head is obtained by using a coupling medium with a dielectric constant of around 20 at 1 GHz (a mixture made of TX-100 and salt water) as used in [54] instead of air. Moreover, as might be expected, choosing a proper coupling medium is particularly important here because it helps to improve the antennas matching to the head’s phantom. Indeed, due to the high attenuation of the different layers of the head, a better adaptation of the antennas helps to have a clear image of the stroke despite this high attenuation, as evidenced in Figure 6c,d that displays the differential field obtained by subtracting the electric field obtained inside the head in the absence of the stroke-affected tissue to that obtained in its presence.

#### 3.3.2. Effect of the Plastic

**Figure 7:**
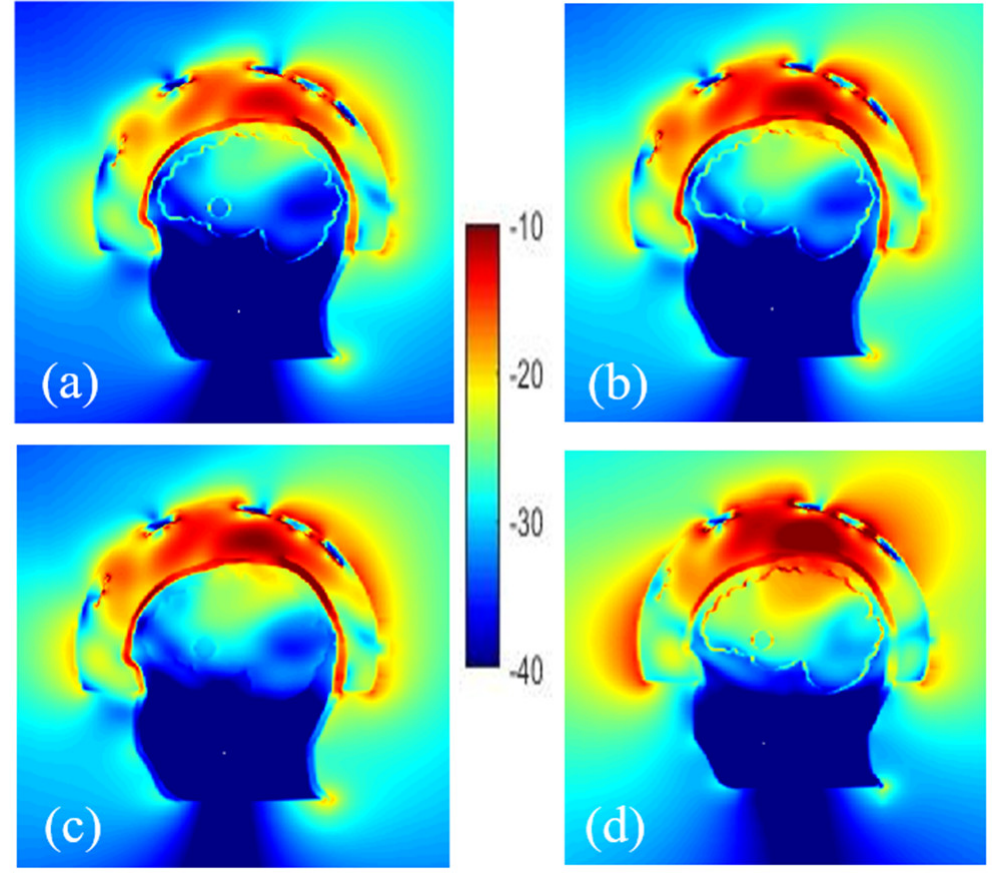
Normalized electric field magnitude distribution inside and outside the head including cavities made of: ABS shell (**a**), without ABS for stroke (**b**), stroke and brain (**c**) and only bone (**d**).

Different configurations are simulated to study the effect of plastic on the field distribution depending on its location, i.e., around the stroke, the stroke and the brain, the bone. Therefore, the cavities of phantom including head, brain, and stroke made of ABS are considered. The other scenario is to consider the containers with the same dielectric properties as the TMM. At first sight, using phantoms made of ABS may not seem practical due to the low relative permittivity of ABS but the results depicted in Figure 7 indicate that removal of ABS does not have a noticeable effect on the E-field distribution inside the brain, except for the skull whose thickness varies from 2 to 8 mm. Thus, an alternative material whose dielectric constant is similar to the bone’s dielectric constant (around 10 at 1 GHz) should be more suitable to print the head external wall. “3D-Prima Conductive ABS” filament seems to be a good candidate [57].

#### 3.3.3. Effect of Phantom Complexity

- Number of cavities

More realistic phantoms can be achieved by designing additional cavities where, for example, different parts of the brain are separated or other tissues such as skin, muscle, and fat are taken into account. Thus, before the experiments, the validity of the phantom can be evaluated numerically by studying the effects of adding new cavities in the simulations. Here, an additional cavity in the lower part of the head, filled with a mimicking mixture for muscle tissues, is considered. In such a way the numerical model corresponds to the second version of the physical phantom of the head shown in Figure 3.

**Figure 8:**
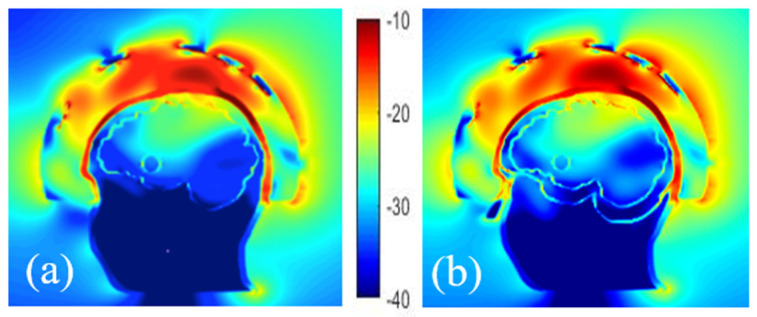
Comparison of the normalized E-field distribution inside and outside of head phantoms with a stroke, composed of 2 cavities (**a**) and 3 cavities (**b**).

On comparing the E-field distribution inside the brain with these two cases (Figure 8), it can be seen that the amplitude of the E-field is similar, which is probably due to the experimental configuration considered (antennas array conformal to the head) while the dielectric properties of the mixture in the lower part of the head seem to have a low effect on the distribution of the electric field in the brain.

- Vessels around a randomly shaped stroke

**Figure 9:**
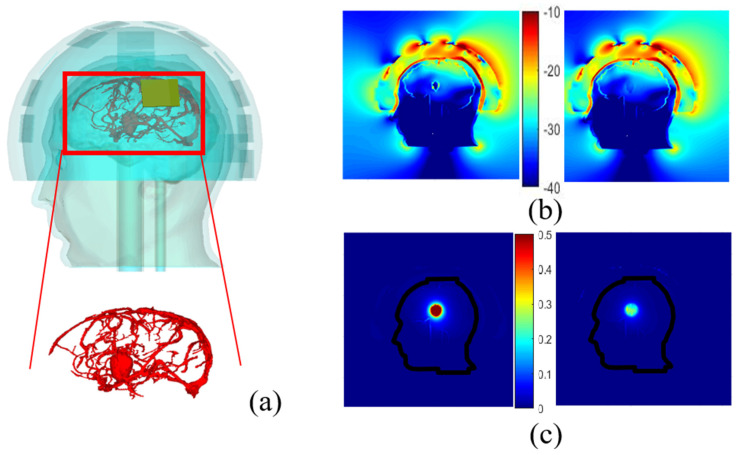
Head phantom including brain vessels and an elliptical stroke inside (**a**). Normalized magnitude of the E- field inside and outside the head by taking blood vessels into account and the elliptical stroke, surrounded by ABS (**b**, left), with no ABS (**b**, right); the corresponding differential E-field (**c**, left) and (**c**, right) respectively.

A more sophisticated numerical head phantom including a brain model that accounts for blood vessels is investigated to check the possibility of recognizing a randomly shaped stroke. The object represents a bleeding case with a height and width of 2.6 cm and 1.8 cm, respectively. The dielectric properties of the blood are set for it and simulations are done with and without a plastic shell, in the presence of the blood circulation. This model, including an elliptical stroke inside, is depicted in Figure 9a, whereas Figure 9b,c displays the total and differential E-field distributions, in the two different cases: with and without the plastic shell. In Figure 9b,c, it appears that the presence of the ABS structure leads to an overestimation of the differential field due to the low dielectric properties of the plastic compared with the TMM around it. Other simulations show that in the given experimental configuration of the system, the vascularization of the brain does not affect the differential distribution of the field, when a stroke is considered. As a result, this quantity ∆E, remains a proper quantity to track the status of the stroke over time since the stroke is still visible and well positioned in the middle of the brain for both cases.

## 4. Conclusions

A method of developing benchmark phantoms for the assessment of microwave devices was presented in this paper, demonstrating that the process previously introduced to develop the GeePs-L2S breast phantom can be used to design other body parts. It has also been shown that Kraszewski’s binary mixing law, like Böttcher’s, can be introduced into the optimization code described in [19], to numerically determine the recipe for mixtures that mimic almost all types of biological tissues. These results were experimentally validated. Changes in the design of the head phantom were performed to adapt the phantom to the microwave imaging system developed in [5] showing that this type of phantom is applicable in any experimental configuration. CST Microwave Studio simulations were performed thanks to the numerical version of the phantoms (STL file). This opens up new avenues for assessing microwave devices and some of them were addressed in this paper. It was shown that by using a more realistic brain cavity inside the head phantom, illuminated by a set of antennas in a helmet such as the one developed in [5], the variation of the electric field distribution due to the existence of a stroke is still visible, especially if the coupling medium is well chosen. It was underlined that the choice of a mixture with a dielectric constant of 23 is an appropriate coupling medium between the antenna helmet and the head, as outlined in [54]. Also, a model to study the effect of blood vessels of the brain was considered, showing that qualitative differential imaging could have potential in this example. We also studied the effect of ABS on the electric field distribution in the head phantoms, which is a controversial subject [56]. We also showed that the presence of plastic shells around the anomalies leads to an overestimation of the differential field, making them more visible. This remark is in agreement with that described in [56]. However, let us recall that a benchmark phantom is first of all an object used to evaluate and compare the performance of different systems. Ten years ago, infinite cylinders were used as the reference [58,59], while today phantoms are anthropomorphic. This constitutes a significant step forward even if there is still room for improvement. Furthermore, the most significant effect of ABS on the distribution of the electric field inside the brain remains that of the outer layer, which is thicker. This means that the effect of the plastic depends on the experimental configuration as well as the phantom under consideration; the numerical-based methodology proposed in this paper allows such investigations. Besides, the progress of 3D-printing technology suggests that low-cost printing of solid structures with dielectric properties similar to those of biological tissues will soon be available. Future works will focus on simplifying the phantom’s meshing in order to reduce the high-computational cost of the microwave imaging algorithms that would use the anthropomorphic models [60].

## Figures and Tables

**Table 1:** Concentration and properties of different TMMs at 1 GHz and 25 °C.

	Mixture Components Given by the Kraszewski and Böttcher’s Binary Laws		Kraszewski–Böttcher and Cole–Cole Dielectic Properties	
Tissue	TX-100 (vol %)	NaCl (g/L)	$\varepsilon_{r}$	σ (S/m)
	Kraszewski–Böttcher	Kraszewski–Böttcher	Binary law–Ref [51]	Binary law–Ref [51]
Brain	34–36	6.8–6.9	41–42	1.0–1.0
CSF	5–6	13.9–14.0	68–68	2.5–2.5
Muscle	21–22	5.5–5.4	54–55	1.0–1.0
Bone	80–75	1.0–2.0	12–12	0.2–0.2
Blood	14–14	9.2–9.0	60–61	1.6–1.6
